# Determination of hemoglobin mass in humans by measurement of CO uptake during inhalation of a CO‐air mixture: a proof of concept study

**DOI:** 10.14814/phy2.13849

**Published:** 2018-09-03

**Authors:** Roberto Falz, Martin Busse

**Affiliations:** ^1^ Institute of Sport Medicine and Prevention University of Leipzig Germany

**Keywords:** Blood volume, CO method, COHb, progressive dosage technique

## Abstract

Measuring hemoglobin mass (Hbmass) using the carbon monoxide (CO) bolus rebreathing method is frequently used in research but has yet to be widely used in the clinical practice. The estimation of an adequate CO bolus may be difficult in patients with unknown Hbmass. In the present pilot study, a progressive inhalation technique for CO that leads to a linear individual adjusted COHb increase was evaluated. Sixteen healthy test subjects participated in the study (preliminary investigation: six; main study: ten). The reliability and validity of the new method were evaluated using multiple measurements of Hbmass with and without a defined blood donation and compared to a CO bolus method. The participants inhaled a CO‐air mixture (CO concentration: 1500 ppm) for a specific breathing duration. The CO uptake and COHb change were determined simultaneously. The typical error (reliability) in the repeated measurements was 2.4% (CI ± 4.7). The mean difference between the new method and the bolus method was 34 g (±41; *P* = 0.026). The measured hemoglobin loss in 490 mL of blood was 74 g (±35), and the calculated hemoglobin loss was 77 g (±4) (mean difference 3 g ± 34; *P* = 0.820). The new method was reliable and valid in a proof of concept study with healthy subjects. The total amount of CO and as a result the COHb increase is individually adjustable. Future studies in clinical settings are needed to determine if the method could be used in disease‐specific pathologies associated with changes in Hbmass.

## Introduction

The estimation of hemoglobin mass (Hbmass) and blood volume (BV) is relevant in the clinic (Christensen et al. 1993; Jones and Wardrop 2000; Fouad‐Tarazi et al. 2007; Ahlgrim et al. 2014; Karlsen et al. 2016) and for exercise physiology. However, currently no method for determination of hemoglobin mass is used frequently in clinical settings. Blood volume changes are often estimated using indirect indicators, such as hematocrit, the hemoglobin concentration. However, concentration‐dependent parameters do not indicate the absolute values.

All methods used to determine the BV are based on the indicator dilution principle. Performing red blood cell counts using ^51^chromium (^51^CR)‐labeled cells is generally accepted as the gold standard of BV measurements (The International Committee for Standardisation in Haematology, 1980). Due to its radioactivity, this method is not widely used. Recently, the carbon monoxide (CO) rebreathing method has been improved several times (Thomsen et al. 1991; Burge and Skinner 1995; Hütler et al. 2000; Schmidt and Prommer 2005; Gore et al. 2006; Siebenmann et al. 2017), and the application of this method has been reported in many studies. The accuracy of the CO dilution method has been shown to be similar to that of the ^51^CR method (Fukui and Shigemi 1989; Ohki et al. 2000). CO binds almost completely to hemoglobin. Therefore, the increase in carboxyhemoglobin (COHb) after the inhalation of a defined amount of CO is inversely related to the Hbmass. Subsequently, the oxygen transport capacity is reduced by the percentage of the CO increase.

The accuracy of the CO method is affected by the complete CO distribution and knowledge of the absorbed CO (Siebenmann et al. 2017), including the possible loss of CO to extravascular tissue or an incomplete mixing in pooled blood and exhalation. An incomplete distribution of CO in the vascular system may lead to an underestimation of the Hbmass (Keiser et al. 2013). Therefore, a reasonable distribution time should be allowed following the CO administration and even more in clinical‐related situations. Complete blood mixing can be assumed by similar COHb values in venous and arterialized capillary (Hütler et al. 2000; Garvican et al. 2010) or arterial blood samples (Garvican et al. 2010). In an open circuit system, a respiratory CO loss may occur, which could result in the overestimation of the BV. Several authors assumed a CO diffusion in the extravascular compartments, particularly with regard to myoglobin (Shimazu et al. 2000; Bruce and Bruce 2003, 2006; Schmidt and Prommer 2005; Prommer and Schmidt 2007; Garvican et al. 2010). However, a relevant diffusion of CO has not been confirmed (Richardson et al. 2002).

In commonly used CO rebreathing protocols the CO is administered as a bolus into a rebreathing curcuit (Christensen et al. 1993; Burge and Skinner 1995; Schmidt and Prommer 2005). Two versions of the CO rebreathing method are used. Burge and Skinner (1995) proposed a 10‐min rebreathing period of a CO bolus diluted in oxygen. In 2005, Schmidt and Prommer introduced an “optimized CO rebreathing method” with only 2 min of rebreathing and inhalation of the whole CO bolus within the first several seconds. The “optimized CO rebreathing method” requires patient cooperation for specific breathing maneuvers. The bolus volume is determined by the patient′s sex, body composition, and fitness level before the test. Thus, the estimation of an adequate CO bolus may be difficult in patients. However, CO‐saturated autologous blood has also been used (Fukui and Shigemi 1989; Ohki et al. 2000; Sawano et al. 2006). After the inhalation of a pure CO bolus, the arterial COHb rapidly increases within the first few seconds (COHb peak) depending on the patient′s absolute Hbmass, which might further reduce the oxygen transport capacity in anemic or hypovolemic patients. The use of a CO bolus in healthy subjects with predictable Hbmass does not result in any symptoms (Siebenmann et al. 2017). In summary, the administration of a CO bolus could include uncertainty regarding the CO‐dosing and the resulting COHb increase, particularly in patient groups where Hbmass is difficult to predict. In addition, rebreathing involves an active participation of the patient and bears the risk of contaminating the closed system.

The objective of this study was to introduce a new progressive dosage technique of CO administration in an open circuit (no rebreathing) and measure the inhaled and exhaled CO amounts during the entire test period. The expected benefit of the inhalation of a CO‐air mixture is a linear increase in COHb (Coburn et al. 1965; Hauck and Neuberger 1984; Tikuisis et al. 1987). The CO uptake was measured breath‐by‐breath according to the difference in the CO concentration between the inspiration and expiration air multiplied by the airflow. The increase in COHb was measured in multiple capillary blood samples simultaneously.

## Materials and Methods

### Ethical approval

This study was approved by the Ethical Committee at the Medical Faculty, Leipzig University (reference number 183‐12‐26092011) and was conducted in accordance by the latest revision of the Declaration of Helsinki. All participants provided written informed consent. The study group (*n* = 16; Table 1) consisted of three females and 13 males. The exclusion criteria included pregnancy, heavy smoking, any reported diseases and women during the menstrual phase.

**Table 1:** Subject characteristics (*n* = 16)

Study arm	Pre‐study	Main‐study
Age (years)^a^	26.7 ± 3.2	26.4 ± 2.5
Height (cm)^a^	176 ± 7	176 ± 9
Body mass (kg)^a^	73.0 ± 10	71.4 ± 12
Gender	6 males	7 males/3 females

a Mean and standard deviation.

### Study design

The **preexamination** included a medical history, height and weight measurement, an electrocardiogram, a pulmonary function test, and a bio‐impedance analysis to determine the body fat and lean body mass.

In a **preliminary investigation**, the CO distribution time during the administration of a low CO‐air mixture (1500 ppm CO, synthetic air: 20% oxygen and 80% nitrogen) and the accuracy of the flow measurement in the breathing system were measured. To determine the CO distribution in the vascular space, the venous and capillary values in six subjects were compared during and after the low CO‐air mixture breathing period. The subjects inhaled a CO‐air mixture for 6 min, followed by ambient air for 3 min. Heparinized capillary blood and venous blood from the hyperemized ear lobe and cubital vein were simultaneously sampled, and COHb was analyzed (ABL80 CO‐OX Flex RiliBÄK, Radiometer Medical ApS, Brønshøj Denmark) immediately before, at min 3 and 6 of the CO breathing period and at minutes 7, 8, and 9 (distribution period).

The **reliability and validity** of the new method were tested by repeated measurements (same subject) of hemoglobin mass with and without a defined blood donation and compared to a reference bolus method. In total, the Hbmass of 10 subjects was measured four times on four consecutive days. The new method was used in test 1, 2, and 4. Test 2 was a modified CO bolus rebreathing method. A blood donation was made before test 4.

#### Reliability

The Hbmass was measured twice using the new method to quantify the reliability within 24 h at the same time of the day (Tests 1 and 2).

#### Validity

The validity was tested by determining the Hbmass on days three and four using a CO bolus rebreathing method (Test 3) and determining the Hbmass after a blood donation of 490 mL at the Institute for Transfusion Medicine of the University of Leipzig (Test 4, new method).

Any blood loss following the first examination was reported by the subjects before the following Hbmass determination was conducted. A significant increase in the Hbmass due to a spleen contraction or the rapid release of reticulocytes in this 4‐day period is not expected (Prommer et al. 2007; Pérez‐Ruixo et al. 2008).

### Test procedure of the progressive CO dosage method (Tests 1, 2, and 4)

High‐purity synthetic air with 1500 ppm CO was inhaled to increase the COHb, followed by three minutes of breathing room air before determining the final CO distribution in the blood. The inhalation and expiration CO concentrations, COHb values, and ventilation were measured throughout the entire test period (Fig. 2).

At least two capillary blood samples were obtained from the earlobe, and the COHb, hemoglobin concentration (cHb), and hematocrit were repeatedly analyzed (ABL80 CO‐OX Flex RiliBÄK, Radiometer Medical ApS, Brønshøj Denmark; measurement principle: spectrophotometry) immediately before the CO‐air breathing step.

**Figure 1:**
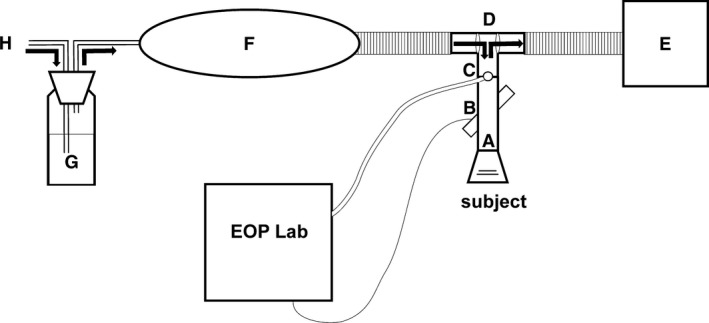
Schematic overview of the breathing system for the progressive CO dosage method; **EOP** **Lab:** Easy‐One‐Pro‐Lab ndd Medical Technologies, Zurich, Switzerland, (A) Spirette and mouth piece, (B) ultrasonic flow meter with handle and USB cable, (C) gas sampling valve and sampling line, (D) patient valve, (E) expiration tube connected to a Douglas bag, (F) reservoir bag, (G) humidifier, (H) connecting tube to the test gas bottle with variable area flow meter (not shown in figure; 1500 ppm CO and synthetic air).

The subjects were seated for 10 min and then connected to the breathing system. The subjects inhaled a CO‐air mixture (test gas 1500 ppm CO in synthetic air: 20% oxygen and 80% nitrogen; Riessner Gase GmbH, Germany; Fig. 1). The nose was closed with a nose clip. A reservoir bag (Fig. 1F) allowed for the individually desired ventilation volume. A humidifier (bubble humidifier, medap Josef Gesson GmbH, Austria, Fig. 1G) was integrated, and the air humidity was approximately 50%. The gas supply was controlled by an adjustable flow meter (DK800/N, Krohne GmbH, Germany; Fig. 1H). The exhaled air was sampled in a Douglas bag (Fig. 1E). The ultrasonic flow/volume sensor (obtained from Easy‐One‐Pro‐Lab, ndd Medical Technologies, Zurich, Switzerland, Fig. 1B) was located between the patient valve and the mouthpiece, which was a part of the disposable hygienic breathing tube of the equipment (Spirette, ndd Medical Technologies, Zurich, Switzerland Fig. 1A). The connection to the patient valve was attached to a sampling valve (Fig. 1C) on the distal part of the breathing tube, thus allowing for simultaneous gas sampling. A research‐software package (WBreath, ndd Medical Technologies, Zurich, Switzerland) was used for the flow‐ and concentration‐data acquisition, storage, and analysis. After the third minute of the CO breathing period, a capillary blood sample was obtained to measure the COHb. If the COHb increase reached 2.5% or higher, the CO‐air breathing time was limited to 6 min. If the COHb increase ranged from 1.5 to 2.5%, the CO‐air breathing was maintained at 8–9 min to ensure a COHb increase of at least 5%. At the end of the CO breathing period, the CO inspiration tube was disconnected from the patient valve. Then, the subjects breathed room air for 4 min. The CO exhalation during the air breathing is assessed and subtracted from the CO uptake. Capillary blood samples were obtained from both earlobes every minute and analyzed.

The flow sensor, CO sensor (nondispersive infrared), and CO_2_ sensor (nondispersive infrared) were automatically calibrated before and after the measurements.

#### Data processing

**Figure 2:**
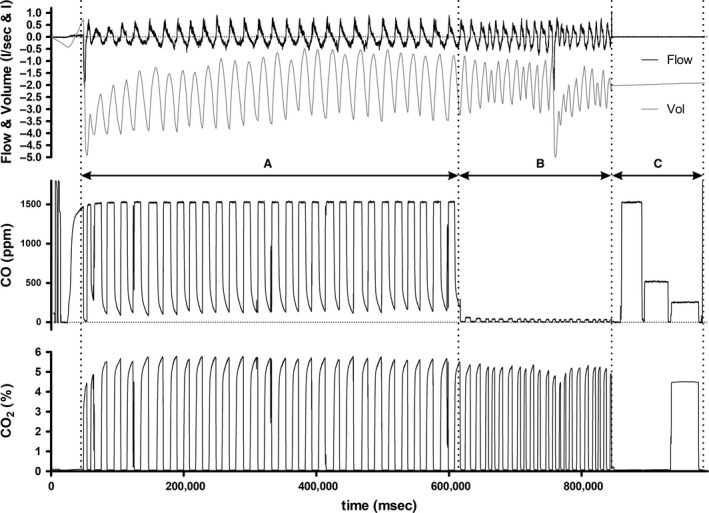
Raw data of flow, CO‐, and CO2‐concentration over time when using the progressive CO dosage method in a single subject. (A) CO breathing period (550 sec); (B) distribution period (236 sec); (C) control measurements of the CO sensor and the CO concentration in the Douglas bag.

“WBreath” software (ndd Medical Technologies, Zurich, Switzerland) and Excel 2007 (Microsoft Inc., Washington, USA) were used for the data processing. The CO uptake during the test and distribution periods was calculated. The raw data (Fig. 2) were processed according to the manufacturer's instructions as follows for each measurement:

#### Calculation of the flow/volume

A BTPS (body‐temperature‐pressure‐saturated) correction of the flow data was calculated. In preliminary experiments, we verified a changed flow geometry in the breathing system. This flow bias was caused due to the patient valve in front of the flow sensor. Therefore, a gain correction of the inspiratory flow data was performed in addition to the BTPS correction in WBreath. To control the flow correction a full exhalation at the beginning and end of the breathing period was used (Fig. 2).

#### Calculation of the CO concentration

A correction for the time delay of the CO signal due to the gas transfer time from the patient valve to the analyzer device was applied; the offset (approximately 90–100 ppm) and linear drift correction (approximately 50–100 ppm CO in 10 min) of the CO raw data, the correction of the CO signal for the influences of the exhaled CO_2_ (cross‐sensitivity of 23 ppm CO per percent CO_2_), and the linearization of the CO data were performed using a device‐specific linearization factor provided by the manufacturer. In the final step, the CO uptake during the entire test was calculated by summing the breath‐by‐breath CO flow over time.

### Test procedure CO bolus rebreathing method (Test 3)

The CO rebreathing method was based on the methodologies of Burge & Skinner (1995) and Schmidt and Prommer (2005). The closed breathing system (not illustrated here) consisted of a tube system, a breathing bag (size: 5 L), and a CO_2_ absorber filled with soda lime (Wenoll System, EMS GmbH, Germany) to prevent CO_2_ accumulation. Furthermore, an oxygen supply (Riessner Gase GmbH, Germany), an oxygen sensor (GOX 100 O_2_ sensor, GREISINGER Electronic GmbH, Germany), and a flow meter (Easy‐on‐PC, ndd Medical Technologies, Zurich, Switzerland) were integrated in the system.

The capillary blood samples were obtained from the earlobe, and the COHb, cHb, and hematocrit values (ABL80 CO‐OX Flex RiliBÄK, Radiometer Medical ApS, Brønshøj Denmark) were repeatedly analyzed immediately before the rebreathing. The subjects were seated for 10 min while connected to the rebreathing system via a mouthpiece, that simultaneously served as a bacterial filter. The nose was closed with a nose clip. Subsequently, the subjects remained connected to the closed system for 15 min, and oxygen was continuously added (0.3–0.6 L/min) and monitored. An individual CO bolus was applied via a 100 mL glass syringe (Fortuna, Poulten & Graf GmbH, Germany) during a deep inhalation (Schmidt and Prommer 2005). Then, the subjects remained connected to the closed system for 15 min. For the CO bolus, we applied 1 mL per kg of lean body mass for women and 1.2 mL per kg of lean body mass for men (99.997%, CO‐Minican, Linde AG, Germany). In preliminary studies, this CO volume resulted in a COHb increase of approximately 5% in women and men. At the end of the rebreathing phase, the subjects were disconnected after the expiration to their residual volume, and the breathing system was closed with a sealing cap. We determined the remaining volume in the rebreathing system (CO_2_ absorber and tubes: 1050 mL), the individual exhaled volume using an ultrasonic flow meter, the individual residual volume (Stocks and Quanjer 1995) and the CO concentration (Fluke CO‐220, Fluke Corporation, USA) in the rebreathing system to calculate the volume of unabsorbed CO. During the CO rebreathing, arterialized capillary blood samples were obtained at 1, 3, 5, 7, 9, 11, 13, and 15 min after the addition of the CO bolus. We used the mean steady‐state COHb value (samples obtained at 9, 11, 13, and 15 min) to calculate hemoglobin mass. A typical error of 1.9% we could verify in own investigations for this method (unpublished data, *n* = 104).

### Calculations:

(1) $$\mathrm{Hbmass}=K\times {\text{CO}}_{\mathrm{calculation}}\times 100\times {\left(\Delta \text{COHb}\times 1.39\right)}^{-1}$$

where

- Hbmass = hemoglobin mass (g)
- *K* = current barometric pressure × 760^−1^ (mmHg) x (1 + (0.003661 × current temperature (Celsius)))
- CO_calculation_ = new method: CO_uptake_ (mL)−(CO_uptake_ × 0.0023 (mL x minutes^−1^) x *t*_respiration_ (minutes))*
bolus method: CO_bolus_ (mL)−(CO_bolus_ × 0.0023 (mL x minutes^−1^) x *t*_respiration_ (minutes))* (− CO_remaining in rebreathing system_ (mL))
- ∆COHb =new method: difference between mean COHb 2/3 min after CO breathing and initial COHb
bolus method: difference between mean COHb in the steady state and initial COHb
- 1.39 =Hüfner's number (mL CO x tHb^−1^)

*correction factor for CO loss (own investigations, unpublished data)

### Statistical analysis

All statistical analyses were performed using SPSS 11.0 (SPSS Inc., Illinois) and GraphPad Prism 4.0 (GraphPad Software Inc., California, USA). Descriptive statistics, including the arithmetic mean and standard deviation (SD), were calculated. The reliability of the method was tested by performing a test–retest correlation analysis and determining the typical error (TE) within the repeated measurements. The limitations of compliance were calculated by multiplying the typical error by 1.96 (95% confidence interval; CI). A Bland–Altman plot was used to graphically present the test differences. A Student′s *t* test (normally distributed data) and Mann–Whitney‐U‐test (non‐normally distributed data) were performed to calculate the differences in the repeated measures. A two‐way ANOVA was used to compare the time course of COHb in venous and capillary blood.

## Results

### Pretest: COHb kinetics

**Figure 3:**
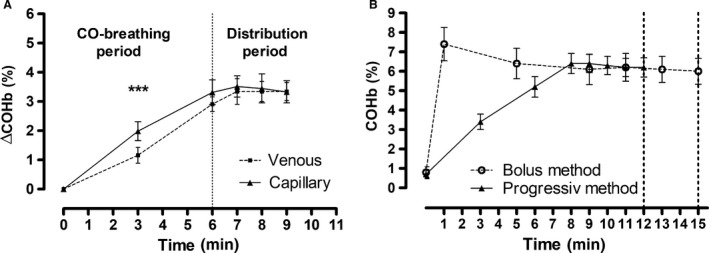
(A) Time course of COHb (mean and confidence interval) in venous and capillary blood during and after the CO inhalation (*n* = 6). The CO breathing time was 6 min, and the consecutive distribution time was 3 min (solid line: capillary blood; dashed line: venous blood). (B) Time course of COHb (mean and confidence interval) when using the new and the bolus rebreathing method (*n* = 10; solid line: progressive CO dosage method; dashed line: bolus method; vertical dashed line at min 12 and 15: end of progressive dosage method and end of bolus method).

The COHb time course showed an approximately linear increase during the CO breathing period (Fig. 3A). However, the venous and capillary COHb significantly differed during the CO breathing time (minute 3: mean difference −0.8% ± 0.3; *P* < 0.001; minute 6: mean difference 0.4% ± 0.3; *P* > 0.05). During the CO distribution time, the venous and capillary COHb concentrations showed no statistically differences (minute 7: mean difference −0.2% ± 0.2; *P* > 0.05; minute 8: 0.1% ± 0.2; *P* > 0.05; minute 9: 0.0 ± 0.1; *P* > 0.05).

### Reliability of the new method (Test 1 vs. Test 2)

**Figure 4:**
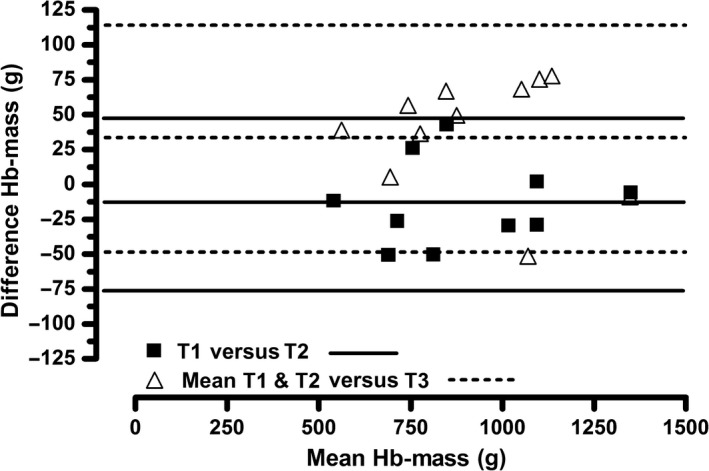
Bland–Altman plot of the average Hbmass in Test 1 versus Test 2 (new vs. new; *n* = 10) and Tests 1 and 2 versus Test 3 (new vs. bolus; *n* = 10) (new vs. new = solid lines: mean difference −13 ± 31 g; 95% limits of agreement 48 to −74 g; new vs. bolus = dotted lines: mean difference 34 ± 41 g; 95% limits of agreement 115 to −47 g).

Figure 4 shows the differences in the Hbmass across the repeated measurements using the new method (■ T1 vs. T2). There is no systematic bias (Fig. 4). The mean difference among the repeated measurements was −13 g ± 31, that is, the mean relative difference was 1.5%. No significant difference (*P* = 0.199) was observed among the repeated measurements of the Hbmass (Table 2).

**Table 2:** Hbmass in tests 1–4 (*n* = 10)

tHbmass [g]	Reliability repeated measurement using new method	Validity new method versus bolus method	Validity repeated measurement after blood donation
Test 1	887 ± 224	893 ± 243#	893 ± 243#
Test 2	900 ± 242		
Test 3 (Bolus)		927 ± 233	
Test 4			819 ± 232
Mean difference	13 ± 31 (*P* = 0.199; ns)	34 ± 41 (*P* = 0.026; *)	−74 ± 35 (*P* < 0.001; **)
Calculated difference	0	0	−77 ± 4 (*P* = 0.820; ns†)

Values are presented as the mean and standard deviation; # = mean of Tests 1 and 2; ns = not significant; * = significant difference between the mean of Tests 1/2 and Test 3; ** = significant difference between the mean of Tests 1/2 and Test 4; † = no significant difference between the measured difference and the calculated difference (mean of Test 1/2 and Test 4 after the blood donation).

A close relationship of *r* = 0.99 was observed between the Hbmasses obtained in Tests 1 and Test 2. The repeated measurements resulted in a typical error (TE) of 22 g (CI: ±43) Hbmass in the entire group or a relative TE of 2.4% (CI: ±4.7).

### Validity (new method compared to bolus method; Tests 1&2 vs. Test 3)

Figure 4 shows the differences in the Hbmass between the tests using the new method (Tests 1 and 2) and the bolus method (Test 3) in a Bland–Altman plot (∆ mean T1 & T2 vs. T3). Systematic error (bias) can be assumed (Fig. 4). The mean difference in the measurements (Table 2) was −34 g ± 41, resulting in a mean relative difference of 3.7% ± 4.4. The Hbmasses calculated using the new and bolus methods significantly differed (*P* = 0.026; Table 2). Nevertheless, a close relationship (*r* = 0.992) was observed between the Hbmasses obtained in Tests 1 and 2 and that obtained in Test 3. The repeated measurements resulted in a TE of 29 g (CI: ±43) Hbmass, corresponding to a relative TE of 3.2% (CI: ±6.2).

### Repeated measurement after blood donation (Tests 1&2 vs. Test 4)

The measured reduction in the Hbmass averaged −74 ± 35 g (*P* < 0.001, Table 2). The real loss of hemoglobin (Table 2) was calculated according to the volume of donated blood and the hemoglobin concentration (blood volume x cHb). The difference between the measured and calculated Hbmass reduction was 3 ± 34 g (*P* = 0.820).

The raw data for baseline COHb and cHb, the increase in COHb as well as the CO volumes bound to hemoglobin are presented in Table 3.

**Table 3:** Initial COHb, ∆COHb, hemoglobin concentration, and CO uptake and test duration in Tests 1–4 (*n* = 10)

	Test 1 (new method)	Test 2 (new method)	Test 3 (bolus method)	Test 4 (new method after blood donation)
Initial COHb [%]	0.7 ± 0.4	0.7 ± 0.5	0.8 ± 0.4	0.7 ± 0.7
∆COHb [%]	5.5 ± 1.1	5.5 ± 0.7	5.3 ± 1.1	6.1 ± 0.8
Hemoglobin concentration [mmol/L]	9.6 ± 0.8	9.6 ± 0.7	9.4 ± 0.8	8.9 ± 0.9
CO uptake[mL]	63 ± 16	64 ± 19	66 ± 17	58 ± 17
Test duration [sec]	628 ± 47	635 ± 46	900 ± 0	614 ± 73

Values are presented as the mean and standard deviation; ∆COHb in the new method represents the difference between the initial COHb and the mean COHb at minutes 2 and 3 after the CO breathing; ∆COHb in the bolus method represents the difference between the initial COHb and COHb steady state (mean COHb at minutes 9, 11, 13, and 15).

## Discussion

This study is the first to use CO uptake from a low concentrate CO‐air mixture to estimate the Hbmass in whole blood. This procedure leading to an almost linear increase in COHb, facilitates the dosing process and reduces the risk of excessive increase in COHb, particularly in patient groups where Hbmass is difficult to predict. By simultaneously monitoring capillary COHb during the CO breathing period using the new method, the COHb increase can be approximately adjusted to 5%, regardless of the Hbmass (this is a reasonable compromise between measurement precision and low impairment of oxygen transport capacity). An increase in the COHb by >5% above the baseline is required for a high measurement accuracy (Burge and Skinner 1995; Alexander et al. 2011; Turner et al. 2014). The progressive CO inhalation can be used without breathing maneuvers and does not require the cooperation of the patient. Use in a ventilator seems to be possible. In addition, all parts exposed to a patient's breath are disposable (spirette) or can be disinfected (patient valve). The use of synthetic air and 1500 ppm CO could be an advantage with regard to possible clinical applications or possible leakage of CO. A medical gas with a higher CO concentration (approximately 3000 ppm CO) is also used to measure the CO diffusion capacity of the lungs (DLCO).

The presented method depends on the precise determination of the CO uptake during the CO breathing period, the exhalation of CO during the distribution period, a complete CO equilibration in the vascular space and a possible CO loss to extravascular tissue. Each of these factors is discussed in the following text.

### Determination of CO uptake and CO loss during the entire test period

The respiratory gas flow was determined using an ultrasound‐based flow meter. The flow meter determines the absolute transit‐time of ultrasonic pulse‐trains (Buess et al. 1986), is considered highly accurate and reproducible (Schibler et al. 2002; Pérez‐Padilla et al. 2006) and is also independent of the gas composition, pressure, humidity, and temperature (Buess et al. 1986).

The Easy‐One‐Pro‐Lab has been successfully used for multiple breath measurements in other studies (Fuchs et al. 2006). To obtain accurate measurements, using the mouthpiece correctly is critical. Particular attention must be paid to the correct lip closure, adequate positioning, tooth position, and prevention of salivation through the Spirette.

To calculate the CO uptake (CO uptake in the breathing period minus the CO lost via exhalation in the distribution period, see in Fig. 2), the flow data were merged with the simultaneously measured CO concentrations during the entire test period. To document the accuracy of the CO concentration measurement, calibrations were performed before and after the test period. In addition, two test gases (real concentration: 519 ± 0 ppm and 1519 ± 10 ppm CO; measured concentration: 513 ± 4 ppm and 1529 ± 18 ppm) in the range of the inspired and expired CO concentrations were measured at the end of each measurement, and a drift correction and a control of the CO concentration measurement accuracy could be performed.

### CO distribution

An incomplete CO distribution could result in the over‐ or underestimation of hemoglobin mass depending on the type of blood sampling (capillary or venous) and time point of blood sampling. In a preliminary experiment, we determined the COHb kinetics in venous and capillary blood during a CO breathing for 6 min to examine the duration of the CO distribution in blood (Fig. 1). In the main study, we used either 6 min or 8–9 min (depending on the first rise in COHb after 3 min) to ensure a COHb increase of at least 5%. Turner et al. (2014) could show that the volume of a CO bolus influences the determination of the Hbmass. Therefore, in further investigations the accuracy of the progressive dosage method depending on the CO‐air breathing duration should be tested. Complete blood mixing can be presumed by similar COHb values in venous and arterial blood samples (Hütler et al. 2000; Garvican et al. 2010). In the distribution period after the CO inhalation, the COHb in the capillary and venous blood was not significantly different. To ensure that a complete CO distribution in the vascular space had occurred, we used the mean COHb concentration at minutes 2 and 3 (sampled at both earlobes to minimize the measurement error) after the CO breathing period to calculate hemoglobin mass. It may be important to consider that the human spleen stores approximately 200 mL of red blood cells (Stewart and McKenzie 2002); this hemoglobin will most likely not be tagged by the indicator dilution methods. This technique would lead to an underestimation of hemoglobin mass. Nevertheless, a loss of precision is not expected (Siebenmann et al. 2017), since the release of stored red blood cells due to a splenic contraction is triggered by sympathoactivation (Stewart and McKenzie 2002), such as heavy exercise. In order to ensure that a complete CO distribution has occurred in clinical‐related situations, such as pulmonary disease or heart failure, we strongly recommend the analysis of venous and arteriallized blood.

### CO loss to extravascular tissues

Possible losses of CO, particularly from myoglobin, are discussed in the literature (Sawka et al. 2000; Bruce and Bruce 2003, 2006; Schmidt and Prommer 2005; Prommer and Schmidt 2007). However, the CO capacity in the extravascular components is much smaller than the hemoglobin capacity (Shimazu et al. 2000). Richardson et al. (2002) did not detect any measurable increase in the CO‐myoglobin concentration as a result of an increased COHb concentration. Burge and Skinner (1995) did not expect a CO loss from the vascular system and, accordingly, used no correction factor. A minor loss of CO (<1 mL after 10 min when COHb is up to 9%) has been described in the multicompartment model (Bruce and Bruce 2003). In our series using the bolus method, we calculated a loss of 0.23% of CO bolus per minute (unpublished data). Due to the linear increase in COHb using the present method, we assume that the mean diffusion gradient between hemoglobin and myoglobin for CO during the entire test period is much smaller than that using the CO bolus method (Fig. 3B).

### Reliability and validity

Regarding the reliability of the present method, typical error (TE) values of 2.4% were calculated in the repeated measurements. Similar studies in the literature include Thomsen et al. (1991): coefficient of variation (CV) 2.5%; Burge and Skinner (1995): CV 0.8%; Hütler et al. (2000): CV 3.3%; Schmidt and Prommer (2005): TE 1.7%; Gore et al. (2006): TE 1.1%; Fagoni et al. (2018): TE 1.35%. Methods using ^51^CR‐labeled erythrocytes have also revealed similar reliability results (Gore et al. 2005; Siebenmann et al. 2017).

The validity of the new method was examined by comparing the new method with the bolus method and performing multiple measurements after a defined blood donation. The Hbmass measurement using the new method was strongly correlated with that obtained using the bolus method (*r* = 0.929), but a significant bias of −34 g (*P* = 0.026) was observed. At a cHb of 15 g/dL, a 34 g Hbmass corresponds to an error of approximately 200 mL of blood.

The results of the repeated measurements after the blood donation (mean error of 3 g) confirm that the new method has an acceptable accuracy in detecting minimal changes in the Hbmass. Overall, the new method has a high reliability and an adequate validity. Based on a calculated typical error of 2.4%, changes in the Hbmass ± 4.7% could be detected with 95% certainty. Assuming a Hbmass of 1000 g, this corresponds to a fluctuation of ±47 g or approximately 300 mL BV (at a cHb of 15 g/dL), respectively.

BV, red cell volume, and their change are often estimated using indirect indicators, such as hematocrit, the cHb or the mean blood pressure. Concentration‐dependent parameters exhibit large within‐day biological variations (Hilderink et al. 2017) and do not indicate the absolute values. An accurate method for measuring the total Hbmass and total BV could provide a potentially useful clinical tool for monitoring BV and fluid and blood transfusion management in surgical and intensive care practices (Fukui and Shigemi 1989; Christensen et al. 1993; Jones and Wardrop 2000). Precise knowledge of Hbmass and BV contribute to the diagnosis of blood disorders, such as anemia, polycythemia vera, or hemodilution, in patients with chronic heart failure (Adlbrecht et al. 2008; Ahlgrim et al. 2014). Karlsen et al. (2016) demonstrated that cardiovascular function and safety indices remained unchanged after using the CO method, and approximately 6% COHb was observed in patients with stable coronary artery disease.

In sports medicine, only minimal changes in BV due to training were demonstrated (Schmidt and Prommer 2008; Eastwood et al. 2009, 2012). Optimal altitude training may cause a mean increase in Hbmass of 1.1%/100 h (Gore et al. 2013). Nonphysiological blood manipulations misusing erythropoietin (EPO) or blood doping may lead to a mean change in hemoglobin of up to 12% (Parisotto et al. 2000). Therefore, multiple repeated measurements of Hbmass in athletes during a year of training could significantly contribute to the detection of blood doping or the effects of high altitude training (Prommer et al. 2008).

In conclusion, we demonstrated that the new progressive CO administration method is reliable and valid in the determination of Hbmass in a proof of concept study with healthy subjects. A low‐concentration CO air mixture can be used without breathing maneuvers, the total dose of CO, and the COHb increase can be individually adjusted. In addition, all parts exposed to the breath are disposable (spirette) or can be disinfected (patient valve). With appropriate further investigations in clinical settings, these first results may indicate that this method has some promise for application in disease‐specific pathologies associated with changes in Hbmass, potentially assisting clinical diagnoses and therapies.

## Conflict of Interest

The authors have no conflicts of interest to report.
